# Handgrip strength is associated with cognitive function in older patients with stage 3-5 chronic kidney disease: results from the NHANES

**DOI:** 10.1038/s41598-024-60869-y

**Published:** 2024-05-06

**Authors:** Jialing Zhang, Peixin Wang, Qi Pang, Shiyuan Wang, Aihua Zhang

**Affiliations:** 1 Department of Nephrology, Xuanwu Hospital, Capital Medical University, 45 Changchun Street, Xicheng District, Beijing, 100053 China; 2 National Clinical Research Center for Geriatric Disorders, Xuanwu Hospital, Capital Medical University, Beijing, China

**Keywords:** Kidney diseases, Neurological disorders, Nutrition disorders

## Abstract

In this study, we aimed to investigate the association between handgrip strength (HGS) and cognitive performance in stage 3-5 chronic kidney disease (CKD) patients aged ≥ 60 years. This cross-sectional study analyzed data from National Health and Nutrition Examination Survey (NHANES) database 2011–2014. Three tests were used to assess the cognitive performance, including consortium to establish a registry for Alzheimer’s disease (CERAD), animal fluency test (AFT), and digit symbol substitution test (DSST). The multivariate linear regression analyses adjusting for confounding factors were utilized to evaluate the association of HGS with cognitive performance. A total of 678 older stage 3-5 CKD patients were included in this study. After adjusting for multiple factors, a higher HGS was positively associated with a higher CERAD-delayed recall and DSST score. In addition, our analysis indicated that HGS probably correlated with better performance of immediate learning ability in male, while working memory, sustained attention, and processing speed in female. HGS may be an important indicator for cognitive deficits in stage 3-5 CKD patients, especially for learning ability and executive function. Further research to explore the sex-specific and domain-specific and possible mechanisms are required.

## Introduction

Chronic kidney disease (CKD) has a major effect on global health, affecting approximately 10% of the global population^1^. Evidence indicated that patients with CKD are at an increasing risk of cognition impairment, which is associated with poor quality of life, high hospitalization rate, and mortality^2–6^. The causative mechanisms are multifactorial, probably due to uremic neurotoxicity, cerebrovascular disease, oxidative stress, anemia and dialysis-related factors^7^.

Handgrip strength (HGS) has been shown to be a reliable and inexpensive marker of nutritional status and skeletal muscle function in patients with CKD^8,9^. Especially, an update Kidney Disease Outcomes Quality Initiative (KDOQI) clinical practice guidelines for nutrition in CKD had suggested HGS as a useful indicator of nutritional and functional status^10^. It has been shown that HGS inversely associated with cardiovascular disease outcomes, all-cause mortality and cardiovascular mortality^11–13^. A recent meta-analysis suggests that HGS may be a risk indicator for poor cognitive outcomes, including cognitive impairment, dementia and Alzheimer's disease^14^. However, limited associations between HGS and preclinical Alzheimer cognitive composite score was showed^15^.

To date, few studies have evaluated the relations of HGS and cognitive function in CKD patients. The aim of this analysis was to investigate the sex-specific associations of HGS with cognitive dysfunction in patients with stages 3-5 CKD.

## Results

### Participant characteristics

Our final analysis consisted of 323 males and 355 females aged 60 years and older. As indicated in Table 1, patients were aged 72.01 ± 6.87 years. A total of 228 and 448 patients reported being a current smoker and having a recent drinking episode, respectively. As for comorbidities, 231 patients had diabetes mellitus, while 534 patients had hypertension. Absolute HGS ranged from 8.3 to 64.9 kg in male and 5.7 to 51.8 kg in female. There was a significant difference between male and female patients in terms of drink, smoke, physical activity, eGFR, hemoglobin, glucose, cholesterol and uric acid. Additionally, the scores of CERAD-WL, CERAD-DR and DSST were significantly different between male and female cohorts.

**Table 1:** Baseline characteristic of the study population.

	Total (n = 678)	Male (n = 323)	Female (n = 355)	P
Age (year)	72.01 ± 6.87	72.18 ± 6.91	71.86 ± 6.85	0.498
Male (%)	47.6			
Race				0.322
Mexican American (%)	4.4	5.3	3.7	
Other Hispanic (%)	6.0	6.8	5.4	
Non-Hispanic White (%)	50.9	46.7	54.6	
Non-Hispanic Black (%)	33.5	35.6	31.5	
Other (%)	5.2	5.6	4.8	
Education				0.045
Less than 9th grade (%)	11.5	13.6	9.6	
9 − 11th grade (%)	16.5	16.4	16.6	
High school graduate (%)	26.3	24.1	28.2	
College or AA degree (%)	26.5	23.2	29.6	
College graduate or above (%)	19.2	28	16.1	
CKD stage				0.298
CKD 3 (%)	92.0	92.9	91.3	
CKD 4 (%)	5.3	4.0	6.5	
CKD 5 (%)	2.7	3.1	2.3	
Smoke (%)	33.7	42.4	25.8	< 0.001
Drink (%)	66.1	80.6	53.7	< 0.001
Diabetes (%)	34.1	35.0	33.2	0.632
Hypertension (%)	78.8	74.9	82.3	0.02
Physical activity				0.01
Inactive (%)	73.9	69.7	71.3	
Active (%)	26.1	30.3	28.7	
BMI (kg/m^2^)				0.068
< 25	34.9	34.7	35.1	
25.0–29.9	25.8	29.6	22.2	
≥ 30	39.3	35.7	42.6	
GNRI	103.17 ± 13.45	102.5 ± 15.97	103.78 ± 10.63	0.958
Hemoglobin (g/dl)	13.29 ± 1.50	13.72 ± 1.57	12.89 ± 1.31	< 0.001
Albumin (g/l)	41.35 ± 3.22	41.36 ± 3.35	41.34 ± 3.10	0.728
BUN (mg/dl)	21.41 ± 9.04	21.64 ± 8.96	21.21 ± 9.12	0.342
Creatinine (μmol/l)	129.71 ± 89.02	144.14 ± 83.97	116.58 ± 91.53	< 0.001
Cholesterol (mmol/l)	4.72 ± 1.22	4.42 ± 1.14	5.00 ± 1.23	< 0.001
Triglyceride (mmol/l)	1.81 ± 1.16	1.80 ± 1.14	1.81 ± 1.20	0.992
Glucose (mmol/l)	6.61 ± 2.92	6.84 ± 3.04	6.41 ± 2.80	0.002
Uric acid (mg/dl)	6.52 ± 1.53	6.64 ± 1.48	6.42 ± 1.57	0.022
eGFR (ml/min/1.73m^2^)	46.34 ± 11.29	48.30 ± 11.16	46.46 ± 11.35	0.01
UACR (mg/g)	169.02 ± 722.16	233.43 ± 904.08	109.88 ± 494.12	0.646
CERAD-WL score	18.19 ± 4.70	17.26 ± 4.28	19.03 ± 4.90	< 0.001
CERAD-DR score	5.58 ± 2.42	5.02 ± 2.37	6.08 ± 2.35	< 0.001
AF score	15.55 ± 5.22	15.51 ± 5.24	15.58 ± 5.22	0.962
DSST score	41.93 ± 16.53	39.16 ± 14.83	44.45 ± 17.58	< 0.001
Handgrip strength (kg)	31.06 ± 10.38	38.49 ± 9.08	24.33 ± 5.96	< 0.001

*AF* animal fluency test, *CERAD*-*WL* consortium to establish a registry for Alzheimer’s disease word learning test, *CERAD*-*DR* consortium to establish a registry for Alzheimer’s disease delayed recall test, *DSST* digit symbol substitution test, *CKD* chronic kidney disease, *BMI* body mass index, *eGFR* estimated glomerular filtration rate, *BUN* blood urea nitrogen.

### Associations of HGS with related factors

Results from Spearman's correlation analysis was shown in Table 2. In both male and female patients, higher HGS was significantly associated with younger age, more favorable physical activity, higher BMI, eGFR, GNRI, hemoglobin and albumin levels, and lower UACR and BUN. In addition, the HGS positively correlated with each cognitive test score, including CERAD, AFT, and DSST.

**Table 2:** Associations between handgrip strength and related factors.

	Total		Male		Female	
	ρ	P	ρ	P	ρ	P
Age (year)	− 0.301	< 0.001	− 0.431	< 0.001	− 0.535	< 0.001
Physical activity	0.270	< 0.001	0.224	< 0.001	0.267	< 0.001
BMI (kg/m^2^)	0.074	0.059	0.191	0.001	0.126	0.022
Hemoglobin (g/dl)	0.331	< 0.001	0.212	< 0.001	0.155	0.0034
Albumin (g/l)	0.151	< 0.001	0.198	< 0.001	0.182	0.001
Creatinine (μmol/l)	0.291	< 0.001	− 0.208	< 0.001	− 0.056	0.292
eGFR (ml/min/1.73m^2^)	0.192	< 0.001	0.256	< 0.001	0.101	0.057
BUN (mg/dl)	− 0.220	< 0.001	− 0.417	< 0.001	− 0.299	< 0.001
UACR (mg/g)	− 0.190	< 0.001	− 0.335	< 0.001	− 0.236	< 0.001
Total cholesterol (mmol/l)	− 0.137	< 0.001	0.088	0.116	− 0.005	0.918
Triglyceride (mmol/l)	0.001	0.983	0.056	0.319	− 0.021	0.695
Glucose (mmol/l)	0.046	0.233	− 0.032	0.566	− 0.074	0.164
GNRI	0.165	< 0.001	0.223	< 0.001	0.022	< 0.001
Uric acid (mg/dl)	0.114	0.003	0.025	0.654	0.121	0.023
CERAD-WL	0.006	0.867	0.183	0.001	0.255	< 0.001
CERAD-DR	− 0.015	0.691	0.124	0.126	0.298	< 0.001
AFT	0.144	< 0.001	0.15	0.007	0.255	< 0.001
DSST	0.087	0.023	0.156	0.005	0.377	< 0.001

*AF* animal fluency test, *CERAD*-*WL* consortium to establish a registry for Alzheimer’s disease word learning test, *CERAD*-*DR* consortium to establish a registry for Alzheimer’s disease delayed recall test, *DSST* digit symbol substitution test, *BMI* body mass index, *BUN* blood urea nitrogen.

### Associations of HGS with cognitive function

The relationships of HGS and cognitive tests were analyzed using multivariable linear regression models (shown in Table 3). In total CKD patients, HGS was positively associated with CERAD-DR and DSST scores.

**Table 3:** Associations between handgrip strength and cognitive function.

	CERAD − WL			CERAD − DR			AFT			DSST		
	Adjusted β (95% confidence interval)	P	P for interaction	Adjusted β (95% confidence interval)	P	P for interaction	Adjusted β (95% confidence interval)	P	P for interaction	Adjusted β (95% confidence interval)	P	P for interaction
Total	− 0.023 (− 0.066, 0.02)	0.293		0.024 (0.002, 0.046)	0.031		0.004 (− 0.042, 0.05)	0.871		0.161 (0.031, 0.292)	0.016	
Sex			< 0.001			< 0.001			0.009			< 0.001
Male	0.090 (0.019, 0.161)	0.013		0.016 (− 0.023, 0.054)	0.426		0.035 (− 0.048, 0.118)	0.405		0.021 (− 0.182, 0.225)	0.837	
Female	0.083 (− 0.029, 0.195)	0.146		0.070 (0.017, 0.023)	0.01		0.112 (− 0.002, 0.226)	0.054		0.520 (0.186, 0.853)	0.002	
Smoke			0.431			0.058			0.279			0.314
Yes	− 0.029 (− 0.106, 0.048)	0.457		0.042 (0.005, 0.079)	0.027		0.042 (− 0.037, 0.122)	0.29		− 0.199 (− 0.46, 0.062)	0.134	
No	− 0.007 (− 0.063, 0.049)	0.799		− 0.005 (− 0.034, 0.023)	0.724		− 0.011 (− 0.073, 0.050)	0.724		− 0.076 (− 0.257, 0.106)	0.414	
Drink			0.463			0.681			0.4			0.369
Yes	0.054 (0.008, 0.100)	0.021		0.043 (0.018, 0.067)	0.001		0.041 (− 0.012, 0.093)	0.133		− 0.161 (− 0.325, 0.004)	0.056	
No	0.023 (− 0.059, 0.105)	0.574		0.003 (− 0.036, 0.043)	0.868		− 0.025 (− 0.109, 0.059)	0.553		− 0.166 (− 0.424, 0.093)	0.208	
DM			0.884			0.678			0.083			0.127
Yes	− 0.011 (− 0.095, 0.074)	0.807		0.012 (− 0.031, 0.054)	0.591		− 0.01 (− 0.096, 0.076)	0.819		− 0.196 (− 0.441, 0.049)	0.117	
No	− 0.034 (− 0.807, 0.019)	0.206		0.044 (0.017, 0.71)	0.002		0.013 (− 0.046, 0.072)	0.662		0.186 (0.008, 0.365)	0.041	
Hypertens − ion			0.541			0.1			0.141			0.347
Yes	− 0.019 (− 0.068, 0.3)	0.446		− 0.020 (− 0.044, 0.004)	0.106		0.005 (− 0.047, 0.057)	0.855		− 0.122 (− 0.279, 0.036)	0.129	
No	− 0.061 (− 0.174, 0.052)	0.288		0.072 (0.009, 0.134)	0.025		− 0.017 (− 0.143, 0.110)	0.792		− 0.322 (− 0.732, 0.088)	0.122	
UACR			0.594			0.706			0.34			0.045
< 30 mg/g	0.065 (0.020, 0.109)	0.005		0.044 (0.020, 0.067)	< 0.001		0.001 (− 0.052, 0.054)	0.957		0.187 (0.025, 0.348)	0.023	
30 − 300 mg/g	0.051 (− 0.043, 0.145)	0.289		0.003 (− 0.042, 0.048)	0.895		0.09 (− 0.006, 0.187)	0.066		0.046 (− 0.255, 0.347)	0.762	
≥ 300 mg/g	0.020 (− 0.210, 0.250)	0.861		− 0.017 (− 0.120, 0.086)	0.743		0.019 (− 0.174, 0.212)	0.843		− 0.316 (− 0.843, 0.201)	0.223	

*AF* animal fluency test, *CERAD*-*WL* consortium to establish a registry for Alzheimer’s disease word learning test, *CERAD*-*DR* consortium to establish a registry for Alzheimer’s disease delayed recall test, *DSST* digit symbol substitution test.

The association of HGS with cognition function in patients with CKD was further stratified by sex, smoke, drink, diabetes, hypertension, and UACR. In male patients, HGS was positively associated with CERAD-WL, while HGS was positively associated with both CERAD-DR and DSST in female patients. Additionally, an interaction between HGS and sex was found (p for interaction < 0.05). Stratified analyses also showed that patients with a higher HGS had better cognitive performances than those with a lower HGS in the subgroups with drink status, without albuminuria and without comorbidities of diabetes or hypertension. However, the association between HGS and cognitive impairment risk was less pronounced in patients with diabetes, hypertension and albuminuria. Notably, no significant interaction effects were observed between HGS and diabetes, hypertension, smoke, and drink status.

## Discussion

Our findings firstly provide evidence for the independent association between cognitive dysfunction and HGS in older patients with stage CKD 3-5. Our research prompts an overall measurement of HGS and cognitive function should be emphasized to assess physical health impairment and cognitive impairment in CKD patients in the clinical practice.

Muscle strength is an important measure of physical fitness and has been associated with all-cause and cardiovascular mortality^16^. HGS has become a stronger predictor of nutritional status^17^, all-cause death, cardiovascular death, and cardiovascular disease in the general population^18–20^. In patients on chronic dialysis, a weak HGS was also suggested to be an independent predictor of all-cause mortality^21,22^ and cardiovascular events^23^. Importantly, emerging data showed that HGS is linked with multiple aspects of cognition^24^, including verbal ability, spatial ability, processing speed, and memory^25^. A prior study reported that HGS was independently related to a declining of cognitive function^26^. Another longitudinal study also clarified that a HGS change was also associated with worse cognitive performance (via mini-mental state examination (MMSE))^27^. In cancer survivors, an increase of HGS was associated with a higher score on both AFT and DSST^28^. To date, there is no clinical studies evaluating the relationships of HGS and cognitive function declines in CKD patients.

Our findings showed that HGS was significantly linked with a delayed learning ability and working memory, assessed by CERAD-DR and DSST, in stage 3-5 CKD patients. From the age over 65 years, a weaker HGS was associated with steeper declines in global cognitive function^29^, especially in memory and language domains in non-CKD without dementia population^30^. After adjustment for possible confounders, low muscle strength was associated with poorer performance in the delayed word recall test, verbal fluency test, and trail making test^31^, which are in line with our findings. The mechanisms contributing to the associations between HGS and cognitive dysfunction in patients with CKD is multifactorial.

Skeletal muscle exercise induces expression of the myokine irisin, which linked to skeletal muscle mass and strength^32,33^. Evidence showed a bridge role between irisin and various neurodegenerative diseases, containing Alzheimer’s disease, Parkinson’s disease, and epilepsy^34^. Irisin level was positively correlated with hippocampal brain-derived neurotrophic factor levels, and hippocampal cell proliferation^35^. One the other hand, HGS may be a biomarker of overall strength, and nutritional status^36,37^. In our study, we also found HGS positively correlated with physical activity, BMI, GNRI, hemoglobin, albumin, and uric acid, and negatively correlated with renal dysfunction. A significant relationship between malnutrition and cognitive dysfunction in the elderly was showed^38^. Additionally, decline of HGS may relate to increasing levels of inflammation, such as tumor necrosis factor-α^39^, and C-reaction protein^40^. A weak HGS was associated with a poorer performance on tests of visual memory, language, and executive function, accompanied with a lower total brain volume^41^. A stronger HGS was significantly associated with increased hippocampal volume in people with major depressive disorder^42^ and lobar brain volumes in patients with Alzheimer's disease dementia^43^. Additionally, lower HGS was associated with smaller whole-brain volume, reduced cortical thickness, and higher white matter hyperintensity volume^15,44,45^.

The association between HGS and all-cause mortality tended to be stronger in women than in men^46^. A sex-specific discrepancies in associations between HGS and cognitive function was also clarified before^28,47,48^. There was a significant difference of cognitive scores between male and female patients, and the interaction of HGS and sex was significant in our study. In our cohort, the percentage of smoking and drinking status were significantly lower in female than in male patients. It is reported that both drink and smoke were risk factors for cognitive impairment^49^. Subgroup analysis also revealed a significant association of HGS and cognition impairment in patients with smoke and drink status. In addition, residual renal function might affect the role of HGS on cognition. Although the interaction of HGS and UACR was not significant in this study, we found HGS was related to cognitive function in CKD patients without albuminuria. Patients with macroalbuminuria probably had a worse residual renal function and more comorbidities. Thus, smoking cessation, alcohol restriction and ameliorating albuminuria is recommended in the early stage of CKD.

Sex differences in domains of cognitive function are documented. Namely, HGS was associated with delayed learning ability (CERAD-DR), sustained attention, processing speed, and working memory (DSST) in female, and was associated with immediate learning ability (CERAD-WL) in male. There were significant associations between HGS and short-term memory, language, and delayed memory only in male^48^. By contrast, HGS could positively predict MMSE scores only in older women, but not in men^50,51^. A sex differences in dementia risk was also reported^52^. Concretely, men had more modifiable dementia risk factors than women, while the sex differences in memory performance were relatively small. The sex-specific difference of cognitive decline might be due to a sex hormone changes after menopause and loss of physical function. A relationship was observed between testosterone levels and HGS^53^, while bioavailable testosterone was associated with processing speed, sustained attention, and working memory^54^.

This study has some limitations to be acknowledged. The including patients from our study were limited to only two annual cycles in NHANES, and most of them were at stage 3 CKD, which could lead to a sampling bias. Our cross-sectional design of the study could not infer a causal relationship between HGS and cognitive impairment. Cognitive tests collected from NHANES may not fully represent overall cognitive function. Future longitudinal studies examining the effects of changes in HGS on multiple cognitive function are warranted.

## Conclusion

Our results confirmed that a higher HGS was associated with a better performance of learning ability for novel information and working memory in stage 3-5 CKD patients. A potential sex-specific relationship between HGS and specific domains of cognitive function was also demonstrated. The neurobiological mechanisms and whether exercise programs to improve muscle strength could prevent cognitive dysfunction in patients with CKD need to be further explored.

## Methods

### Study design and participants

The National Health and Nutrition Examination Survey (NHANES) is a cross-sectional survey fielded by the U.S. National Center for Health Statistics. NHANES uses a complex stratified, multistage, probability cluster sample designed to represent the U.S. population^55^. Written informed consent was obtained from all the participants. The NHANES protocol was reviewed and approved by the National Center for Health Statistics Research Ethics Review Board. 19,931 participants 60 years of age and older were sampled. A total of 678 patients met the following inclusion criteria: (1) data available on cognitive data; (2) stage 3-5 CKD [estimated glomerular filtration rate (eGFR) < 60 ml/min/1.73m^2^]; and (3) the grip test performed on both hands. EGFR was calculated using the CKD- Epidemiology Collaboration formula, and staged CKD according to the eGFR-based Kidney Disease: Improving Global Outcomes (KDIGO) classification: CKD stages 3 (eGFR 30–59 mL/min/1.73 m^2^), 4 (eGFR 15–29 mL/min/1.73 m^2^) or 5 (eGFR < 15 mL/min/1.73 m^2^)^56^. Patients lacking sufficient sociodemographic data, including age, sex, race, body mass index (BMI), smoking and alcohol consumption, regular exercise, education level, and relevant laboratory data were also excluded.

### Handgrip strength assessment

The grip test component measured the isometric grip strength using a handgrip dynamometer (Model T.K.K.5401). The participant was asked to use one of the hands to squeeze the dynamometer as hard as possible, and the test was then repeated for the other hand. Each hand was tested three times, alternating hands between trials with a 60-s rest between measurements on the same hand. HGS was measured in kilograms (kg) in each hand, and the maximum value was recorded as the final HGS for subsequent statistical analysis.

### Cognitive assessment

Cognitive function was assessed by the word learning and recall modules from the consortium to establish a registry for Alzheimer’s disease (CERAD) test, animal fluency test (AFT), and digit symbol substitution test (DSST). The CERAD test consists of three consecutive learning trials and one delayed recall to evaluate the immediate and delayed learning ability of new language information (memory subdomain)^57^. AFT examines categorical verbal fluency, a component of executive function. The task in AFT asked participants to name as many animals as possible in one minute, where the total number of named animals was summarized as the test score^58^. DSST examines sustained attention, processing speed, and working memory^59^. Participants were asked to draw as many symbols paired with numbers as possible within 2 min in the 133 boxes.

### Covariates

Data on age, ethnicity, education level, BMI (kg/m^2^), physical activity, smoke status, drink status, hypertension (yes, no), diabetes (yes, no), and multiple laboratory index were extracted as covariates. The sociodemographic factors were collected from the participants. A modified Global Physical Activity Questionnaire was used to measure physical activity^60^. The metabolic equivalent (MET) scores of each type of physical activity (including vigorous work-related activity, moderate work-related activity, walking or bicycling for transportation, vigorous leisure-time physical activity, moderate leisure-time physical activity) were calculated. Physical activity was divided into inactivate and activate according to the median level of MET. BMI was calculated as weight in kilo-grams divided by the square of height in meters. We classified BMI as normal < 25 kg/m^2^, overweight 25–30 kg/m^2^, and obese > 30 kg/m^2^. Geriatric nutritional risk index (GNRI) is an effective indicator for assessing nutritional risk according to the formula: GNRI = [1.489 × albumin (g/L)] + [41.7 × (body weight/ideal body weight)]^61^. Urine albumin to creatinine ratio (UACR) was calculated to assess the renal function (normal: UACR < 30 mg/g, microalbuminuria: UACR 30–300 mg/g, and macroalbuminuria: UACR ≥ 300 mg/g). Blood samples were collected from the vein of each participant in the morning after overnight fasting for at least 8 h.

### Statistical analysis

Statistical analysis was performed with IBM SPSS Statistics 23. Descriptive characteristics were expressed as mean ± standard deviation (SD) for continuous variables and percentage for categorical variables. We used Mann–Whitney’s U-test to compare differences between two groups with non-normal distribution, and Chi-square test for classified variables. Multiple linear regression analyses were used to estimate the β and 95% confidence intervals (CI) for the association between HGS and cognitive function with adjustment of all covariates. Adjusted variables included age, education levels, hemoglobin, albumin, cholesterol, triglyceride, uric acid, eGFR blood urea nitrogen (BUN), blood glucose, BMI, GNRI, UACR and physical activity. The variance inflation factor (VIF) was used to evaluate multicollinearity between independent variables. When the VIF was > 10, the corresponding variables were considered as significant multicollinearity and eliminated. Subgroup analyses were performed by linear regression model, and the interaction between HGS and covariates on the risk of cognitive deficit was also analyzed. Statistical significance was set as p < 0.05.

### Statement of ethics

All participants submitted written informed consent and were approved by the NCHS Research Ethics Review Board (Continuation of Protocol #2011–17).

## Data Availability

The datasets generated and analyzed in the present study are available on the website of NHANES datasets 2011–2014 (https://wwwn.cdc.gov/nchs/nhanes).
